# Factors influencing the generation of teachers’ emotions

**DOI:** 10.3389/fpsyg.2024.1392965

**Published:** 2024-07-26

**Authors:** Lihua Lai, Wenjiang Shi, Yi Xie, Jin Zhao

**Affiliations:** ^1^School of Education, Guangzhou University, Guangzhou, China; ^2^Shenzhen Polytechnic University, Shenzhen, China

**Keywords:** teachers’ emotions, influencing factors, analytical framework, generative mechanisms, phenomenology of education

## Abstract

**Background and aim:**

The teaching profession plays an important role in shaping individuals’ lives, with teachers performing complex emotional labour. The management of emotions is an integral part of teachers’ professional work, and it is essential to clarify their emotional experiences and the generating of their emotions within a specific cultural context.

**Methods:**

Based on a phenomenological approach and the use of anecdotal texts, this study examined six common emotional states among teachers, including happiness, guilt, worry, fear, annoyance, and anger, along with the emotional experiences of two specialised categories of teachers, class supervisors, and pre-service teachers. The factors influencing teachers’ emotions and their generative mechanisms were analysed.

**Results and discussion:**

This investigation found that key influences on teachers’ emotions stem from factors within the teachers’ themselves, the contextual nature of their work, and sociocultural dynamics. Drawing on the analytical frameworks of emotional geography theory, ecological theory of human development, and the ecosystem model of teachers’ emotional interactions, the study constructs a model highlighting the generative mechanisms of teachers’ emotions, and in which three systems are reflected.

**Conclusion:**

Teachers’ personal attributes are in the direct area of the model and directly govern the formation of their emotions, while their work context consists of a transitional area in emotion formation and the sociocultural system acts as the latent band influencing emotion development. The mechanism model helps us to understand and recognise teachers’ emotions and to explore their pedagogical implications.

## 1 Introduction

Education constitutes a complex system (Jacobson et al., 2019) that is essential to the holistic development of an individual throughout their life. The profession places important demands on teachers, and this line of work calls for significant emotional labour (Isenbarger and Zembylas, 2006; Evans et al., 2019; De Ruiter et al., 2021). Emotional labour is a pervasive aspect of teacher’s professional practice (Burić, 2019). Teachers’ emotions impact both their practice and the quality of education students receive. They often encapsulate and reflect the educator’s views on the teaching profession and life in general. The work of teachers is extremely complex, as they need to engage with diverse students as well as navigate intricate educational practices (Keller et al., 2014; De Ruiter et al., 2021). The unique attributes of the profession present many opportunities and challenges but also subject teachers to considerable work-related pressure and emotional issues, which are exacerbated by anxiety in the current educational environment (Chen, 2016). Therefore, the distinct nature of the teaching profession continually tests the emotional intelligence of teachers.

The management of emotions is an important aspect of the teaching profession. It affects the development of educational reform. While some studies establish a relationship between teachers’ emotions and teaching effectiveness (Van Veen and Lasky, 2005; Hodgen and Askew, 2007; Zembylas, 2007; Chen, 2019), little consideration is given to the layers of emotions experienced in the pursuit of school objectives (Van Veen et al., 2005; Chen, 2016). Educators’ emotions affect their teaching and learning practices as well as the achievement of school goals. The identification, appropriate expression, and management of teachers’ emotions not only contribute to the achievement of school goals, but also affect teachers’ well-being, beliefs, behavior, and various facets of their lives (Mevarech and Maskit, 2015).

Teachers’ emotions are not arbitrary; rather, they result from the interactions between many factors that are easily overlooked. Although several studies have investigated teachers’ emotions concerning their work (Nias, 1996; Hargreaves, 2001), a systematic understanding of factors influencing teachers’ emotions is still lacking (Van Veen and Sleegers, 2006). Moreover, although there have been relevant studies on the generation of teachers’ emotions, such as Hargreaves (2001)’ emotional geography framework, the existing frameworks may not fully accommodate the emotional experiences of teachers within the Chinese context. Therefore, it is essential to construct a model to understand the generating of teachers’ emotions tailored to the Chinese context. The objective of this study is to construct a model that elucidates the mechanisms of teachers’ emotional generation. The discussion above motivates this study to address the following questions:

1. What are the key factors affecting teachers’ emotions?
2. How do these factors interact and collaborate to shape emotional responses?

An in-depth analysis of the influences on teachers’ emotions and the underlying mechanisms governing their genesis will facilitate a deeper understanding of teachers and the teaching profession.

## 2 Literature review

### 2.1 The pivotal influence of teachers’ emotions

Teachers’ emotions are a pivotal factor influencing their teaching practices and professional growth, often undervalued in educational reform discourse (Reio, 2011). As a crucial agent in the implementation of educational reform, teachers navigate a multitude of complexities throughout the reform process. The emotional dimension of teaching is integral to its quality, significantly impacting teachers’ self-efficacy and pedagogical strategies (Coppola et al., 2012). Saunders (2013) highlighted the spectrum of emotional responses teachers experience during educational reform, particularly during professional development, and how these emotions could influence the integration of innovative instructional methods. Educational reform is a collaborative and systemic endeavor involving interaction with various stakeholders, such as administrators, colleagues, students, and parents. Teachers’ emotions can serve as a critical lens for understanding educational reform. Teachers’ emotional experiences are closely linked to their daily work and life contexts. Qualitative research evidence provides valuable insights into understanding teachers’ emotions. Di Martino and Sabena (2011) highlighted the relationship between teachers’ emotions and professional development by analyzing the relationship between pre-service teachers’ subject affiliation (as students) and their emotional attitudes toward teaching the subject. Teachers’ emotional experience can affect their teaching methods, class management, and interactions with students, which in turn can influence learning attitudes and academic performance. Coppola et al. (2013) argued that teachers’ emotions can either facilitate or hinder their adaptation to and engagement with reform initiatives. Therefore, educational reform strategies should address the emotional well-being of teachers and aim to reduce negative emotional impacts to foster teachers’ growth as exemplary educators. Despite recent efforts to explore teachers’ emotions, a coherent and comprehensive framework for understanding teachers’ emotions during educational reform is still needed (Sutton and Wheatley, 2003). It is necessary to gain a deeper understanding of how the context in which teachers operate influences their emotions, which would promote the enhancement of teaching practices and the professional growth of teachers.

### 2.2 Framework for analysing factors influencing teachers’ emotions

Hargreaves (2005) proposed the theory of emotional geography as a framework to examine the relationship between teachers’ emotions and educational change. This theory comprises five dimensions: sociocultural geography, moral geography, professional geography, political geography, and physical geography. To ensure the effectiveness of educational changes and policies, administrators need to consider the emotional factors impacting teachers (Hargreaves, 2005). Teachers’ emotional geography is affected by personal, school-related, and sociocultural factors (Hargreaves, 2005). The theory provides a good classification framework for the analysis of teachers’ emotions. Wang and Cheng (2011) further categorised these factors into three levels. First, personal factors include aspects such as personal beliefs, career stage, age, perceptions of one’s abilities, identity, and continuous learning ability. The second level involves school factors, including school culture, micro-politics, leadership style of the headmaster, professional learning opportunities, students’ responses, parental involvement, material support, teacher workload, and time. The third level pertains to socio-economic and cultural factors, encompassing the socio-economic and parental backgrounds, local education policies, curriculum reforms, globalisation, and localized competition. Hargreaves’ “emotional geography” theory provides an analytical framework for clarifying and examining the factors influencing teachers’ emotions. This framework helps in understanding and recognising teachers’ emotions, facilitating the correct handling of their emotional roles in curriculum reforms and pedagogical changes, and ultimately, the enhancement of education quality.

Bronfenbrenner (1979) put forward an ecological theory of human development, where people and the meso-environment in which they live are referred to as a system. He classified this system into five levels, ranging from small to large and from inside to outside, including microsystems, meso-systems, exosystems, macrosystems, and the chronosystem, which is consistent throughout (Cross and Hong, 2012). Sun and Lee (2014) adopted a human ecology perspective to analyse the formation mechanism of teachers’ emotions. They emphasised the crucial role of interactive influence in the process of teachers’ emotion formation, arguing that emotional experiences evolve through the interaction between the individual and their environment. They stressed that this interaction is not only influenced by personality but is also inseparable from the external environment’s impact. The environment includes physical space as well as interpersonal, cultural, organisational, and institutional aspects. Emotions result from the interaction of the individual with the environment, and teachers’ professional practice encompasses the emotional dimension (Van Veen and Sleegers, 2009). Thus, teachers tend to invest themselves in their work, integrating their personal and professional identities. As a result, teaching becomes a pivotal factor for their self-esteem and self-actualisation. The specific nature of the teaching profession determines the inherently emotional labour-intensive nature of teachers’ work (Isenbarger and Zembylas, 2006), which is then closely related to both the development and well-being of children and teachers. Therefore, a comprehensive understanding of teachers’ emotions requires an in-depth exploration and analysis of teachers’ pedagogical practices.

Scholars have conducted comprehensive research on the factors influencing teachers’ emotions, which primarily focuses on individual constraints and human-environment interaction. In terms of individual factors, it is mainly believed that gender, work seniority, work experience, self-efficacy, and beliefs significantly impact teachers’ emotions (Bascia and Hargreaves, 2000; Jiang et al., 2019; Burić et al., 2020). Concerning interactions between people and their environment, it is widely accepted that teachers’ emotions arise from their interactions with teaching or classroom events, as well as their interpersonal relationships (Cross and Hong, 2012; Mevarech and Maskit, 2015). Scholars have studied teachers’ emotions in terms of their generation, with notable frameworks including Hargreaves (2001)’ emotional geography framework, Van Veen and Sleegers (2009)’ socio-cognitive framework, and Pekrun and Linnenbrink-Garcia’s model for analysing the causal cycle of teachers’ emotions (Reinhard and Lisa, 2014). While these analytical frameworks are important references for elucidating the influence of teachers’ emotions and their generative mechanisms, the Chinese context, characterised by locally nuanced teacher roles such as class supervisors and pre-service teachers, necessitates a more appropriate framework for analysis. Therefore, constructing a local framework for analysing teachers’ affective factors becomes essential to address the appropriateness of existing frameworks in the local context.

### 2.3 Interactive analysis of teachers’ emotions in a phenomenological perspective

Research on teachers’ emotions can be typically divided into three dimensions based on the characteristics of the research focus. In the first dimension, researchers frame teachers’ emotions as internal psychological characteristics of individual educators, and they primarily aim to establish the value of teachers’ emotions in education (Nias, 1996). In the second dimension, researchers shift their focus to teachers’ emotions within the realm of social relations, actively paying attention to the relationship between social dynamics, policy changes, and teachers’ emotional experiences (e.g., Isenbarger and Zembylas, 2006). In the third dimension, investigations encompass the sociocultural dimensions of teachers’ emotions, focusing on the shaping, transformation, and resistance of emotions within the context of power dynamics and emotional norms (Schutz and Zembylas, 2009). Given the complex and interactive nature of educational activities, which combine rationality and emotion, attention must be paid to the intricate relationship between teachers’ emotions and educational practices. Canadian scholar Van Manen (1982), adopting a phenomenological approach to pedagogy, extensively studied the emotional experiences of teachers and students, providing very useful research on pedagogy.

Van Manen (1982) outlined a phenomenological approach to assessing pedagogical practice. Phenomenological research involves grasping the nature of phenomena directly through intuitive exploration while suspending foresight (Smeyers, 2018). Teachers’ emotions can be understood as an educational phenomenon, characterized by interaction, process, and complexity. The study of teachers’ emotions necessitates delving into the specific context of teachers’ lives, focusing on the emotional experience of pivotal moments. By conducting an ethnographic study of lived experience, collecting anecdotal writing, and recording experiences through anecdotes, a deeper understanding of teachers’ lives and professional experiences can be attained. A nuanced examination of the variety of experiences in teachers’ professional practice activities allows for a more comprehensive understanding of teachers’ emotions.

Phenomenology serves as an important method for studying teachers’ emotions by immersing into the context where these emotions occur. This approach helps the understanding of the complexity of the factors involved, allowing for the exploration of the causes influencing the generation of emotions and the logic of their interactions. Factors affecting teachers’ emotions exist both explicitly and implicitly within the interaction between individuals and society. Studies ranging from microsystems to macro-contexts, and from individual teachers to sociocultural changes, conducted with a phenomenological path, provide important insights for constructing a localised framework to analyse the interactions of teachers’ emotions in a specific context.

## 3 Methodology

This research adopts a qualitative research orientation, and the choice of research method is determined by the research question (Hammarberg et al., 2016). Qualitative research is concerned with exploring the world through meaning and understanding rather than relying solely on numerical data, thereby providing a more in-depth response to questions of “what,” “how,” and “why” (Lakshman et al., 2000). The choice to adopt a qualitative research approach in this study is primarily attributed to the complexity and implicit nature of teachers’ emotions, necessitating a research method capable of delving into and comprehending the emotional experiences of teachers in the workplace. Qualitative research permits investigators to understand the life experiences of participants from their perspectives (Strauss and Corbin, 1998), a method characterized by its high degree of flexibility and adaptability.

The phenomenological approach is a specific technique within qualitative research methods. This study utilizes a phenomenological approach by collecting personal narratives from teachers regarding their teaching practices to capture the essence of teachers’ emotions. Written data allows respondents to remain anonymous, which is essential for safeguarding their freedom of expression without the apprehension of fulfilling the researcher’s expectations or biases (Di Martino and Sabena, 2011). This method aids in revealing the underlying structures and personal significance of teachers’ emotions, thus offering a more enriched and nuanced perspective for understanding the factors that influence teachers’ emotional experiences.

### 3.1 Research program

To construct a model elucidating the mechanisms of teachers’ emotional generation, it is essential to conduct an in-depth exploration of the key influencing factors of teachers’ emotions. This research primarily utilizes interviews and written anecdotal texts to collect data. Initially, participants voluntarily engaged in the study with the understanding that they may withdraw at any time. Subsequently, participants are requested to write anecdotal texts or respond to the researcher’s questions, such as describing their experiences of happiness, guilt, and other emotional states during the process of teaching practice, detailing the sequence of events, and articulating their feelings. These questions aim to provide a comprehensive account of the participants’ emotional states. Upon obtaining the texts, a phenomenological approach is applied for a line-by-line reflection to further understand the true nature of teachers’ emotions. Finally, an inductive analysis method is employed for data analysis.

### 3.2 Data collection

The focus of this study is on the six common emotional states of teachers, including happiness, guilt, worry, fear, annoyance, and anger, as well as the general emotional experiences of class supervisors and pre-service teachers. These six categories relate to common feelings and experiences, typically encountered in teachers’ daily educational activities. Class supervisors were specifically chosen as participants due to their unique role in the Chinese educational context. Given that teachers, in general, are complex emotional labourers, class supervisors are the most intricate in managing emotions within educational settings. Studying the emotional experiences of pre-service teachers serves as a useful tool for understanding the pathways of teachers’ professional development from the early stages onward.

In this study, over 30 teachers were invited to participate in interviews and write textual anecdotes. It was primary and secondary school teachers who volunteered to participate in the study. The participants were asked to recall experiences involving the six emotional states, recounting the events as they happened and offering detailed descriptions how they felt at the time. Qualitative data for this study was mainly collected between 2020 and 2022, resulting in a total of 108 anecdotal texts capturing teachers’ emotional experiences. The focus of this study is the analysis of these texts.

### 3.3 Data analysis

A clear and detailed data analysis process is essential for conducting rigorous and credible qualitative research (Bingham, 2023). Inductive analysis is a feature and advantage of qualitative research. Based on the collected textual data, this study has employed the inductive analysis method. It adopts a theoretical and practical approach to construct a mechanism for the generation of teachers’ emotions.

The data processing and coding analysis of the data in this study unfolded in three main steps:

1. Phenomenological questioning involves an examination of the data through a sentence-by-sentence reflective analysis, which enabled the understanding of the reality of the pattern of teachers’ emotional experiences. For example, we summarised the text numbered “XF-2-1″ in Table 1 as “inner satisfaction: experiencing student growth.” This was followed by open coding to determine sub-themes related to impact factors, e.g., identifying the influence of the teachers’ emotion pattern “inner satisfaction: experiencing student growth” as “student development” (see Tables 1, 2);
2. The sub-themes related to impact factors were then compared and inductively coded to form secondary codes, i.e., the main categories of the genres. For example, “gender, work seniority, work experience” were categorised as “individual characteristics” (see Table 3);
3. Finally, selective coding was carried out to distill the factors influencing teachers’ emotions according to the framework of personal attributes, work context, and sociocultural levels (see Table 3). During the process of data analysis, members of the research team independently coded the information, subsequently comparing and analyzing the coding results on multiple occasions to arrive at the final analytical outcomes.

**Table 1:** Open coding processes for factors influencing teachers’ emotional experiences (selection).^a^

Teachers’ emotions states	Sub-themes related to impact factors	Patterns of teachers’ emotions	Anecdotal texts
Happiness	Student Development	Inner satisfaction: Experiencing the growth of students	He coyly turned to me and said, “What does 5 × 1/5 equal?” Amidst the outcry of my classmates, I praised him loudly, “I like his honesty and bravery. He is not being hypocritical, and I can tell from his answer today that he is a smart kid. At least for this lesson, he understood it. If you all have time, you can help him catch up on his knowledge, and he’ll be great.” The classroom was quiet at that moment, and everyone cast approving glances at him. From then on, there was another pair of focused eyes in the math class. Sometimes smiling, sometimes helpless, but always filled with a thirst for knowledge. Isn’t this what we expect as teachers? (XF-2-1)
Guilt	Professional Mistake	Frustration resulting from blind use of punishments	Thus, somehow I got really angry and unconsciously hit my hand hard on the desk. There was a “bang” sound, and because the desk surface was very thin, the sound was loud. I was startled, and the classroom immediately quieted down. But only I knew that my hand was vaguely hurting. At the same time, I also noticed their surprised eyes. The students immediately sat down, and while talking students also immediately turned around, staring at me. Suddenly, I regretted it. Could I have controlled the class better? Indeed, I should not have been so angry that the little children were intimidated by my toughness. Next, the new lesson did not go as well as I had hoped, as the atmosphere felt duller and I was frustrated. I felt embarrassed. I had thought that being angry would produce good results, but I did not expect it to make things worse. Before I got angry, some students liked to make eye contact with me. However, when I got angry, their eyes wandered and I felt they were deliberately avoiding my gaze. After class, a girl who is usually well-behaved whispered to me, “Do you know that you were so fierce just now! We were all scared by you.” I was at a loss for words and secretly thought to myself that I would never do that again and that I needed to learn more from other teachers’ teaching experiences to make up for my own shortcomings. (NJ-2-1)

a Due to space constraints, only selected codes are presented.

**Table 2:** Open coding process for factors influencing class supervisors and pre-service teachers’ emotions (selection).^a^

Teacher status	Sub-themes related to impact factors	Patterns of teachers’ emotions	Anecdotal texts
Class Supervisors	Class Management	Management of classroom affairs: Emotions hovering between hope and despair, and pain and care	On the 15^th^ of December, when it was snowing outside, I told the children to turn off the lights, and close the doors and windows in the classroom. The school closed early that day because of the snow, and when I rushed back from outside, I saw that the doors were unlocked and the lights were not turned off, so I criticised them angrily. The next day, on December 16^th^, it happened to be my birthday. As soon as I got home, I received a phone call from a study committee member saying that there were some students in the class who were making a lot of noise and that I should come over and take care of them. I went to the classroom angrily. Some of the students who were usually the troublemakers stopped me and told me to go to the office to talk about it. About ten minutes later, I returned to the classroom and pushed the door open, just as a card fell on my hand and the whole class sang “Happy Birthday.” I saw a big cake on the podium, and they were holding candles and singing the birthday song together. I realised that they had bought the cake themselves and decorated it in the classroom. That scene really touched me! Even though we do not pay much attention to birthdays, the children were very attentive; perhaps they had a conscience and knew that it was difficult for the teacher to accompany them every day in their studies, so they prepared a special birthday present for me. This is one of the most touching events in my relationship with my pupils. (BZR-1-1)
Pre-service Teachers	Community Culture	Relationships with neighbouring communities: From being strangers to friends	The restaurant owner’s wife is very talkative, and the owner is very friendly. Every time we went to eat, we would chat and make some irrelevant jokes. The young son in the restaurant owner’s family was our joy, we always carried him to the dormitory to play, and the owner also let his daughter follow us to go shopping. Their friendly attitude led us to experience a sense of belonging, of being accepted. There was also the owner of the barber shop, who was worried about his son’s poor grades and wanted us to tutor him. We were touched by his eager and trusting gaze and felt a sense of responsibility and mission as teachers. (SX-7-1)

a Due to space constraints, only selected codes are presented.

**Table 3:** Selective coding of factors influencing teachers’ emotions.

Core factors	Main category	Sub-themes related to impact factors
Teachers’ personal attributes	Individual characteristics	Gender (3), work seniority (3), work experience (4)
	Educational beliefs	Educational achievement (2), professional attitude (1), educational justice (2)
	Professional identity	Student development (14), Concerns from students (2), recognition by superiors (1), trust of parents (2), professional value (1), lack of rewards (1), professional image (1), professional identity (3)
	Emotional intelligence	Educational resourcefulness (4)
	Professional quality	Professional mistake (7), professional prestige (1), professional quality (9), management experience (2), professional skills (3), coping skills (1), management skills (1), professional knowledge (1), student management (1), class management (2)
Work context factors	Organisational climate	Support of colleague (1), colleague motivation (2), internship life (3)
	Institutional norms	Management system (2), administrative system (1)
	Interpersonal interactions	Teacher-student interaction (3), teacher-colleague relationship (1), teacher-leader relationship (1), teacher-parent relationship (1), teacher-advisor relationship (6), teacher-student relationship (6), teacher-school member relationship (3), pre-service teacher teammates relationship (4), family-school relationship (3)
	Educational resources	Many chores but lack of resources (1)
	Physical environment	Teaching environment (1), pre-service teacher practice environment (1)
Sociocultural factors	Curriculum reform culture	Emotions in the context of curriculum reform (1), class teaching (1)
	Family education	Family-school collaboration (1)
	Community culture	Community culture (2)

## 4 Results

Through a comprehensive analysis of the factors influencing the six types of teachers’ emotional experiences and the nuanced experiences of class and trainee teachers, this study identified thirteen primary factors influencing teachers’ emotions. Based on the findings and subsequent coding analysis of the data, it is posited that the factors affecting teachers’ emotions can be categorised into three overarching levels, including teachers’ personal attributes, work context factors, and sociocultural factors.

### 4.1 Teachers’ personal attributes

Teachers’ personal attributes include a variety of dimensions, and this study found that in terms of personal attributes, teachers’ emotions are mainly due to their gender, work seniority, work experience, educational beliefs, professional identity, emotional intelligence, and professional quality.

#### 4.1.1 Teachers’ individual characteristics

The research found that teachers’ individual characteristics, including aspects such as gender, work seniority, and work experience, obviously shape the emotional landscape of teachers. Teachers’ emotional experiences were found to emerge from the interplay between their individual characteristics and the environment, with influences stemming from factors like gender, work seniority, and work experience. In terms of gender, female teachers were found to be more sensitive to their inner emotions. One teacher in the study, concerned about her pupil’s homosexuality, used avoidance strategies to cope while hoping to avoid traumatising them. Another teacher delicately described her feelings: “I did not dare look at my students, firstly because I was afraid they would see me crying, and secondly because I was afraid I would not be able to control my tears anymore if I saw them” (XF-3-2). The stereotype associating women with more emotional expression than men is partially confirmed in some studies, particularly in relation to negative emotions (Givon et al., 2023). Yin (2007) argued that male teachers, facing rejection by patriarchal ideology, are reluctant to openly express their emotions in professional scenarios, including teaching or education reform. Female teachers are perceived as less professional because of their focus on emotions. These observations underscore notable differences between male and female teachers in terms of emotional experience, emotional regulation, and emotional perception. Gender characteristics emerge as an important dimension affecting teachers’ emotions.

Concerning work seniority, this study found that young teachers were more prone to anxiety. When faced with a complicated situation, some participants expressed their unease, making statements such as: “I’m just a young teacher in my twenties, so I do not have any experience in dealing with such things and I cannot do anything about it” (YL-7-1), and, “I am worried and under great pressure about how to lead a class, especially with children from the urban–rural transition area who lack good family guidance” (YL-10-1). Another said: “When I first finished school and taught a class, I often felt helpless and worried” (BZR-3-1). A teacher who had just started giving online classes during the epidemic noted: “I panicked that the internet was unstable, that my mind could not work at all, and that the language would not flow” (KJ-1-1). First-time teachers in this study seemed to be easily angered by children who were in a lot of trouble. One noted: “When I first started working in kindergarten, I used to get so angry at the children that I would break out in anger. Why cannot they understand what I’m saying? It’s really disempowering” (FN-1-1).

While some studies indicate that a teacher’s tenure in the profession is considered to have a significant positive effect on the deep active regulation strategy of emotions (Zhou, 2020), our examination of work experience reveals that even veteran teachers grapple with new environments: “As a veteran teacher with 30 years of teaching experience who is about to retire, I am interested in the new way of teaching, such as online teaching, but filled with great anxiety” (KJ-2-1). Teachers who are new to the profession may face additional challenges related to class management, “Why are other teachers so methodical? What on earth am I supposed to do? I was thinking that I’m such a failure, I cannot really teach, and I’m miserable and discouraged because I do not have a method” (YL-10-2). Reinhard and Lisa (2014) analysed the factors influencing novice teachers’ emotions and concluded that they are job-related, with teaching ability and school workload shaping their sense of teaching efficacy and professional expectations. Notably, misbehaviour among students is more likely to provoke negative emotions such as fear, anger, worry, depression, irritation, stress, frustration, and disappointment in novice teachers (Reinhard and Lisa, 2014).

In sum, this study found that teachers’ individual characteristics, such as gender, work seniority, and work experience, vary from person to person and are important factors influencing teachers’ emotions.

#### 4.1.2 Educational beliefs

Teachers’ educational beliefs influence their attitudes. This study revealed instances where some teachers had physically punished students. While the occurrence of spankings may appear rooted in momentary lapses of judgement, a deeper exploration of the issue reveals the problem may be rooted in teachers’ beliefs. In this study, some teachers were affected by parents’ neglect and lack of responsibility for students’ homework, impacting their own attitudes towards students’ homework. This, in turn, manifested in a perfunctory approach to correcting homework, suggesting a potential lack of insight into the complexities of education and a lack of strong educational beliefs to guide their actions. Individuals’ career choices often reflect their fundamental life beliefs, and for teachers, the commitment to teaching becomes an important manifestation of their high ideals as educators.

Teachers, with their passion for education and heartfelt care for their students, are often able to successfully navigate the difficulties and pressures of educational practice, finding profound joy in teaching. One participant said: “The students’ performances are excellent and our joint efforts are rewarded. At this time, I really want to hug each one of them. I think they are very sweet, and I hope they will always be so sweet” (XF-1-1). Despite complaints and the hard work entailed, many teachers persist in the teaching process, experiencing its rewards. One teacher said: “I took over this notoriously challenging class (poor classroom manners, grades, and hygiene) that no one wanted to teach. Now that I’ve taken it over, I’m determined to do a good job with it” (XF-4-1). If teachers do not care for their students from the bottom of their hearts and do not maintain a love for education and a firm belief in education, they will not be able to perform well, even with high levels of knowledge and professionalism.

#### 4.1.3 Professional identity

Our study found that primary and secondary teachers had different perceptions of their professional identity. There were differences in educational expectations between the two groups. Secondary teachers felt upset, helpless, embarrassed, and confused when their subjects and teaching practices were not recognised or respected by students. One teacher expressed: “One student said geography was not on the test, so why learn it? This arrogant tone made me both angry and helpless, and I felt that my dignity as a teacher had been challenged” (YL-14–1). The teaching profession is not insulated from societal utilitarian culture, and teachers of all subjects want to be valued, respected, and recognised for their educational efforts. In our study, we found that some teachers had gained a lot of respect from their students, which had surprised them and positively activated their professional identity. Professional identity is rooted in the emphasis on the “individual self” involving continual choices, identification, and identity construction related to the “social self.” The stronger the teachers’ identification with their work and emotional involvement, the more likely they are to perceive their work as important in their lives, and foster positive subjective emotional experiences.

#### 4.1.4 Emotional intelligence

Teachers can manage their emotions better if they have a high level of emotional intelligence. A teacher described the experience of happiness when invited a student struggling with mathematics to the podium to answer a question related to an easier method of multiplying rational numbers. The teacher keenly observed the shift in the class’s emotional atmosphere and the student’s nervousness, given that the student did not know how to answer the question. The teacher skillfully diffused the situation with encouraging words and praised the student’s honesty in admitting not knowing the answer. This incident fully demonstrated the teacher’s sensitivity and ability to navigate emotional dynamics, and their actions ultimately led to a significant change in the student. The educator noted: “Isn’t this what we expect from teachers?” (XF-2-1). Empirical studies show that the higher the emotional intelligence of a teacher, the better the teacher can deal with incidents and achieve better outcomes (Taxer et al., 2019). Educators with higher emotional intelligence exhibit clearer emotional perception, enhanced communication skills, and a greater ability to meet the demands of their work, ultimately leading to higher teaching efficiency.

#### 4.1.5 Professional quality

This study found that professional quality plays an important role in the formation of teachers’ emotions. Professional quality refers to the deep and unique knowledge, skills, and inner qualities that people gradually develop through professional education or practical training. In a particular instance in this study, a teacher felt inadequate and nervous when he encountered difficulties in subject matters in class due to insufficient preparation. He did not know how to support students in their attempts to understand the challenging matter. The teacher said: “Who knows, every minute and every second of this lesson has been so agonising and difficult for me” (NJ-11–1). Ultimately, at the end of the lesson, the teacher hastily presented the answers to the students. Teachers feel ashamed and guilty for failing in their professional responsibilities, experiencing professional guilt for not preparing and failing to deliver a good lesson. The significance of professional quality is evident, serving as their core competence. Those with good professional quality are better able to identify and control their own emotions, and to guide students effectively in their learning process. However, a teacher with a lack of professionalism might often feel agitated and plagued by negative emotions when faced with unexpected events.

### 4.2 Work context factors

The professional work context in this study refers to the primary workplace of teachers, the environment in which they teach and manage their work, and where their emotions are generated. Teachers’ work contexts are complex and diverse. This study found that the organisational climate, institutional norms, interpersonal interactions, educational resources, and the physical environment of the work context are important factors in influencing teachers’ emotions.

#### 4.2.1 Organisational climate

The organisational climate of the school has a significant impact on teachers’ behaviors and feelings. Organisational climate reflects a shared perception of the work environment. A harmonious and progressive climate is likely to stimulate teachers’ motivation and enthusiasm, thus promoting active engagement and improved performance. Conversely, within a poor and dysfunctional organisational climate, teachers may struggle with heightened psychological pressure, loss of sense of initiative at work, increased frustration, and diminished sense of achievement (Tian and Li, 2006). In this study, a novice teacher participating in a language teaching and research competition felt apprehensive because of his lack of experience; however, his confidence grew rapidly after receiving encouragement and help from colleagues. He said: “When I was apprehensive, my colleagues encouraged me like amiable family members, and their words were like reassurance pills” (XF-5-1). This teacher was ultimately successful and highly rated in the competition. This is a good example of a positive impact of the organisation, reflecting a climate that supports and motivates teachers to make progress. A positive organisational climate nurtures teacher development, building confidence and fostering mutual benefit. However, workplaces often host negative climates, where teachers are treated poorly and disrespected, and are unable to voice concerns or take action.

#### 4.2.2 Institutional norms

The norms and school management system do not have a direct impact on teachers’ emotions, but they do set behavioral expectations for teachers and constrain their behavior. Institutional norms delineate the rules and processes governing all dimensions of the organisational structure, like decision-making, implementation, monitoring, and feedback processes. These norms establish rules for the deployment, use, and management of organisational resources such as people, property, materials, time, and information. They serve as a system of guidance and discipline for individuals (Wu et al., 2010). The normative, democratic, and scientific nature of school institutional management can either restrict or enhance teacher autonomy, affecting their work and emotions to varying degrees. For example, during interviews, teachers talked about the clocking system and salary deductions for even a minute of lateness. They noted how this management practice could cause great distress to teachers. Teachers in the study said: “I have to go to the principal to ask for a leave, and the granting of the leave depends on the principal’s mood, so I sit and wait, not knowing what to do” (BY-1-2). To facilitate the management of their school, some administrators tend to objectify teachers, i.e., treat them as objects to be controlled and observed. These actions suggest that some schools are losing their humanity and flexible orientation. Harsh institutional norms contribute to an excessively strict workplace environment that fosters negative feelings among teachers.

#### 4.2.3 Interpersonal interactions

Teachers’ interpersonal relationships are an important part of their professional development and a pivotal factor in generating teachers’ emotions. These relationships include interactions with all members of the school community, including students, colleagues, leaders, and parents. In this study, class supervisors are important actors who have often had to navigate relationships with students, colleagues, leaders, and parents, often experiencing a range of complex emotions in their interactions. One class supervisor highlighted: “As a class supervisor, I would like to work with regular teachers, not just good teachers, because regular teachers will take time. I can understand my colleagues and it’s easy and brings me relief” (BZR-4-1). Mutual understanding between colleagues can provide teachers with a sense of peace, even when they are in challenging situations. Further, love and understanding from students generate feelings of empathy from teachers, recognition by superiors at the school boosts their confidence and motivation, and trust and respect from parents give teachers a sense of value in education.

In this study, we found that effective parent-teacher communication facilitates teaching and administrative work, contributing to students’ academic performances. However, there were also cases reported where teachers and parents did not communicate well, and there were differences in their educational and classroom management philosophies. When participating teachers sought support from parents to help students with problems, they found issues with parenting methods. For example, in schools for migrant workers’ children, some parents are faced with challenging schedules and believe that education falls primarily under the responsibility of the school and teachers, which impacts their communication with the school or teachers. Some parents take a harsher approach to education, which is not the best way to educate their children. This poses a challenge for teachers involved in family-school cooperation.

#### 4.2.4 Educational resources

Educational resources are an important source of emotional impact on teachers. The lack of educational resources leads to an increased workload for teachers. In this study, certain teachers expressed annoyance due to the encroachment of their part-time administrative duties on their teaching time. One educator said: “We have too much to do here. I start work at 7 am and do not even get a break for lunch. I’m busy until the end of the day” (BY-2-1). Another noted: “In addition to teaching, I’m also the president of the teachers’ union, so I have to do things on both sides, and if I’m busy with administration, I cannot concentrate on teaching, so I obviously feel overwhelmed. Even though I have six years of experience, I find that my teaching standard may not be able to keep up with those teachers who have only been teaching for three or four years” (BY-3-1). In schools facing a shortage of educational resources, a dearth of teaching staff exacerbates the workload for educators, compelling them to wear several hats. Constrained by limited manpower, teachers are burdened with various chores in addition to teaching, leading to dissatisfaction. Teachers lack motivation and complain about the increasing difficulties in carrying out their work. These negative feelings among teachers are caused by the unequal distribution of educational resources and the insufficient availability of high-quality resources. Therefore, the reasonable and fair use of educational resources, alongside the creation of better quality educational materials, is important for improving teachers’ emotional satisfaction and overall happiness at work.

#### 4.2.5 Physical environment

The poor physical environment had a significant impact on teachers’ emotions. The school environment serves the most important site of school life for both teachers and students, offering an important avenue for experiential learning. Transformed and shaped by the collaborative efforts of teachers and students, the school environment assumes an informal role in the cultivation of morals that cannot be achieved by the curriculum. The hidden curriculum, embedded in this environment, serves as a continual source of learning material, fostering diverse experiences and insights for teachers and students. During our study, some teachers complained about the poor school environment, noting that the offices and classrooms were smelly and hot, which significantly affected their mood. A substandard working environment detrimentally affects the physical wellbeing, learning, and work efficiency of teachers and students. Such conditions can make teachers feel depressed, suppressing their passions and originally positive sense of well-being and fostering feelings of irritability, impetuousness, and helplessness. However, sometimes under limited conditions, a positive atmosphere can emerge, as one pre-service teacher claimed: “The school where we did our placements had limited conditions, and could not provide adequate dormitories or offices. Thus, our placement team squeezed into dormitories together, ate together in the cafeteria, and worked in a temporary office, so we all bonded extraordinarily well” (SX-6-1).

### 4.3 Sociocultural factors

Sociocultural factors profoundly influence human beings in many ways, with cultural influences being both pervasive and deeply rooted. In the context of studying teachers’ emotional influences, these factors play a crucial role in influencing teachers’ educational ideals, professional identity, professional development, and interpersonal interactions. Views on life and career by teachers everywhere are shaped by their broader social and cultural context. Specifically, this study found that cultures surrounding curriculum reform, family education, and community culture had a potential impact on teachers’ emotions.

#### 4.3.1 Curriculum reform culture

Curriculum reform plays an important role in regulating and influencing teachers’ emotions. Curriculum reform affects the entirety of the educational landscape and, indeed, the broader social system. This reform, along with the administrative culture of education, undoubtedly exerts a profound emotional impact on teachers, who are subject to a number of detailed educational policy requirements and multiple institutional norms. Teachers are monitored and evaluated from various sources to check whether their practices are in line with the requirements of the curriculum reform. Some teachers claimed: “The school’s requirement of us teachers is that students get good grades and have high-scoring classmates. This situation is contrary to my original intention, so I am worried about my future career. In our secondary education, especially in vocational high schools, can we continue to judge a child’s merits this way, based on grades?” (YL-9-2). The focus on good grades alone in schools represents a regressive culture that instills anxiety in teachers committed to curriculum reform. Teachers can be blamed and punished if their behaviors do not meet expectations (Zhang, 2007). The systems related to curriculum reform, teacher evaluation, and teacher training all have an impact on teachers’ identity, and therefore, influence their emotions (Luo and Yu, 2016).

#### 4.3.2 Family education

Teachers often feel pressurised by parents’ misconceptions of education and the cultural atmosphere, and are unable to cope with parents and students, with interactions leading to feelings of anxiety and fear. In Chinese families, especially those with a single child, parents often show heightened concern about their child’s education. As a result, they treat it as a kind of investment, resulting in the placement of higher demands on teachers. In some under-resourced schools, there are cases where exhausted parents, tired from work, lack the time to look after their children. In this study, we found that many parents send their children to school and take it for granted that the school should take full responsibility for their children’s education, relegating their parental responsibilities to the background. Indiscriminate or non-existent signatures in the “family-school contact book,” incompletion of homework assigned by the class WeChat group, and a lack of communication and collaboration with the teachers become signs of parents’ disregard for cooperation between family and school. These actions also serve as an indication of problems related to views on education in the family. Parental perceptions of education constitute a significant influence on students and teachers. It hinders the work of teachers, which in turn affects their emotions.

#### 4.3.3 Community culture

Our study found that the community in which teachers live has a significant influence on their emotions. The community in which teachers live is another important context that unconsciously and subconsciously influences their sense of community values and other factors. When positive, it can effectively nurture teachers’ spiritual growth, renewing their courage during professional development, freeing them from anxiety and fear, and encouraging a sense of calm and relaxation. “The boss of the restaurant is very talkative, and the owner is very friendly, which makes us feel a sense of belonging, of being accepted (SX-7-1),” said one of the pre-service teachers. Then there was the owner of the hairdressing salon who was worried about his son’s poor grades and wanted us to tutor his son. His eager and trusting eyes touched us and we felt a sense of responsibility and mission as teachers (SX-7-1). Another trainee teacher said: “Sharing and listening to each other in this simple and peaceful community made life and nature more connected” (SX-7-2). A good regional educational ecology creates a community environment supporting teachers’ spiritual growth on a macro level, building a symbiotic platform for mutual assistance. It creates a harmonious community atmosphere, providing fertile ground for each teacher’s personal development, and empowering them to conquer their fears and anxieties.

## 5 Discussion

**Figure 1:**
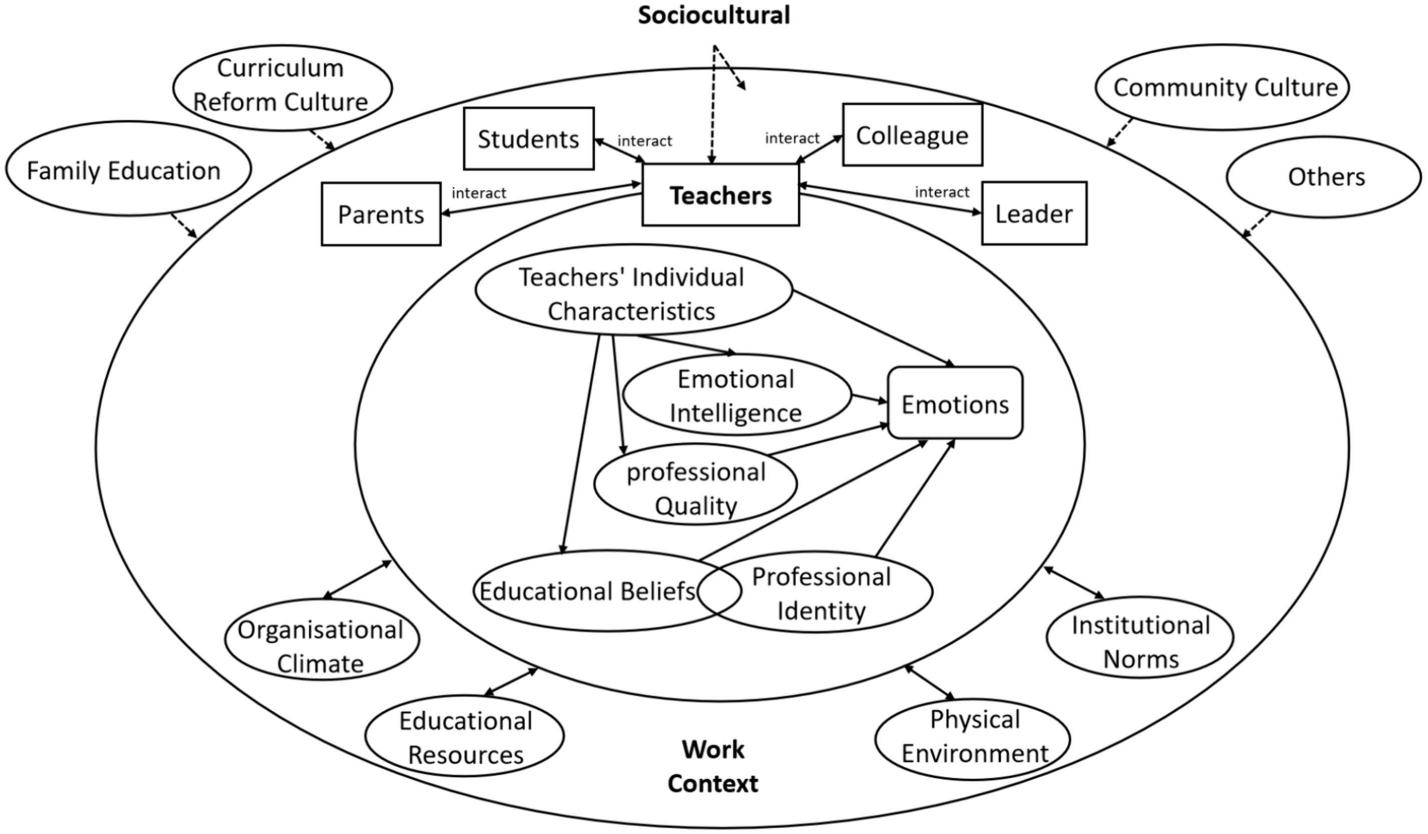
Mechanism model elucidating the generation of teachers’ emotions.

With the objective of constructing a model that elucidates the mechanisms of teachers’ emotional generation within the Chinese context, we employed qualitative research methods, utilizing the writing of anecdotal texts to collect data. Through the process of coding, we identified thirteen key factors that impact teachers’ emotions and are significant determinants in the occurrence of teachers’ emotional responses. Upon analysis, the study revealed that these key factors can be categorized into teachers’ personal attributes, work context factors, and sociocultural factors, which are interwoven and reflect the dynamics of teachers’ emotional generation. Teachers’ personal attributes are in the direct area of the model and directly govern the formation of their emotions, while their work context consists of a transitional area in emotion formation and the sociocultural system acts as the latent band influencing emotion development. The mechanism model constructed in this study, as depicted in Figure 1, captures the factors leading to emotional responses among teachers within the educational environment and portrays the dynamics of teachers’ emotional experiences. This model holds significant importance for understanding the emotions of teachers within the educational context in China.

### 5.1 Teachers’ personal attributes: the direct governing system of the formation of teachers’ emotions

Teachers’ emotions are psychological states arising from the interplay between individual teachers and their environment, influenced by internal factors specific to the teachers themselves. Teachers’ personal attributes influence how they perceive and react to the educational environment and, as identified in our findings, serve as the cornerstone of teachers’ emotional responses in the educational context. Emotions connect the microsystem of teachers (Sun and Lee, 2014). The emergence of teachers’ emotions is directly related to events, but it is not the event itself that dictates the ebb and flow of teachers’ emotions. Rather, it is the teachers’ understanding and reaction to these events that shape their emotional responses. Personal Attributes affect the formation of teachers’ emotions, as shown in Figure 1. Key contributors to the formation of teachers’ emotions include demographic variables, educational beliefs, professional identity, emotional intelligence, and professional quality. Demographic variables such as gender, seniority, and work experience have a direct impact on teachers’ emotions. Male and female teachers experience different emotional “triggers,” leading to distinct emotional responses. Gender-related emotional differences may reflect variations in one or more components of their emotional experience and expression (Gard and Kring, 2007). Further, increased age and work experience lend to more maturity and rationality when facing inconsistency and emergencies. The findings of this study are consistent with Yuan’s study that demographic variables, like gender, age, education, teaching experience, and position, have a significant effect on teachers’ emotional intelligence (Yuan, 2019). In addition, it is shared with other research that teachers’ individual knowledge, educational beliefs, professional identity, experience, and competence in teaching practice are related to emotional understanding, ultimately shaping teachers’ emotions (Zembylas, 2007; Cubukcu, 2013).

Teachers’ individual factors are important subjective influencers of their emotions, and their professional choices serve as important manifestations of their educational beliefs. Strong educational beliefs and a robust sense of professional identity are intertwined, mutually reinforcing one another. Teachers with sturdy educational beliefs and professionalism, and who strongly identify with the teaching profession, tend to exhibit greater maturity. They possess the ability to accurately perceive themselves, readily recognise their emotional states, and deal with unexpected events or conflicts in their lives with a rational mindset, resulting in smoother emotional states. Conversely, teachers lacking a profound belief in education, perceiving it merely as just a job, find it difficult to truly understand the meaning of teaching and nurturing people. This lack of understanding diminishes their sense of responsibility for teaching and their overall happiness. Consequently, these educators tend to become more susceptible to various emotional disorders.

Teachers’ emotions are manifest within a broader context, influenced, constrained, and propelled by both internal and external factors. On an individual level, teachers are their own primary governance system, with the work context then also serving as an influence. The dynamics of teachers’ interactions with students, colleagues, leaders, and parents collectively contribute to the fluid changes in teachers’ emotions. Within the teachers’ internal system, their individual characteristics, educational beliefs, professional identity, emotional intelligence, and professional qualities are the most critical factors influencing teachers’ emotions. Notably, educational beliefs and professional identity exhibit overlapping characteristics, resulting in their effects becoming intertwined. Together, these factors form the teacher’s microsystem, which is the key area from which teachers’ emotions are generated.

### 5.2 Teachers’ work context: the transition zone for the formation of teachers’ emotions

Teachers’ emotions are mainly generated in the work context, and teachers’ emotional experiences within the specific work context are usually generally unfold during the process of teaching, managing, and interacting with different subjects. Factors in the work context impact teachers through their internal system. The work context, depicted in the middle circle in Figure 1, serves as the transition zone for the formation of teachers’ emotions. Emotional experience is generated from interactions between individuals and their environment, shaped not only by the internal characteristics of the individual but by the dynamic interaction with the external environment. Here, the term “environment” extends beyond physical space, to encompass interpersonal, cultural, organisational, and institutional dimensions (Sun and Lee, 2014). The work context, as a transitional zone for emotional formation, is a complex interplay of organisational climate, institutional norms, interpersonal interactions, educational resources, and the physical environment in teachers’ workplaces.

Within the work context, both teachers and their counterparts in interpersonal interactions, including students, colleagues, and parents, are affected by the organisational climate, institutional norms, educational resources, physical environment, and interactions between teachers and different subjects. This work context serves as an important field for teachers to engage with different subjects, at various times every day, through teacher-student interactions, and relationships with colleagues, leaders, and parents. Pre-service teachers must be in close contact with their supervisors and with team members, while the work of class supervisors is even more complex. In these intricate relationships, the relationship between teachers and students stands out as the fundamental interpersonal relationship, while relationships with colleagues, leaders, and parents are also essential. Teachers, as complex emotional labourers due to the special nature of their work, are required to maintain a long-term emotional equilibrium, keeping their spirits high, and always showing optimistic, positive, friendly, pleasant, and calm emotions. In the face of various challenges, teachers need to carry out appropriate emotional work.

The teachers’ work context acts as the transition zone for emotional generation, particularly prone to causing emotional outbursts in specific interaction contexts. The levels of the work context and teachers’ personal qualities mutually influence and interact with each other. When analysing the work context factors that influence teachers’ emotional generation, it is important to clarify the interaction between these factors and teachers’ emotional experiences. Various measures can help improve teachers’ work situations, including creating an equitable organisational atmosphere, creating an open dialogue environment, building a harmonious learning community, formulating a reasonable and standardised work system, ensuring a humane leave policy, implementing a fair evaluation mechanism, establishing a reward system, and promoting a harmonious and comfortable teaching environment. These measures can contribute to the promotion of positive moods among teachers and foster a positive cycle of interactive relationships.

### 5.3 Sociocultural systems: the subtle zones of teachers’ emotional formation

The sociocultural systems, encompassing the curriculum reform culture, family education concepts and styles, and community culture, represent the subtle zones of teachers’ emotional formation, as shown in the outermost circle of Figure 1, constituting an open system. Sociocultural influences are macro factors that impact the formation of teachers’ emotions, acting as a “hidden factor” that influences teachers’ emotions in an infiltrative manner. Our findings suggest that elements at this level do not directly affect teachers’ emotional experiences, but the social context, ideology, and culture of educational reform will affect teachers’ emotions by influencing their thoughts, values, attitudes, and actions. The influence of social culture on teachers’ educational ideals, professional identity, career development, and interpersonal interactions is not only subtle but also deep-rooted. As researchers have argued, culture is one of many environmental factors that influence teachers’ well-being, job satisfaction levels, and job stress levels (Hepburn et al., 2021). It can be said that teachers’ perspectives on life and the profession are everywhere and implicitly sociocultural. It is also the cultural representation of the emotional generation of teachers. Teachers’ emotions reflect the interaction between the individual and social culture.

However, the cultural factors that influence the formation of teachers’ emotions extend beyond those listed in this study, some even more subtle and open. So the outermost level of this model, as shown in Figure 1, is an open system. As Hargreaves points out, the socioeconomic and cultural factors affecting teachers’ emotions include the complexities of local education policies, globalisation, and local competition (Wang and Cheng, 2011). Their influence on teachers’ emotions is often invisible but strong, potentially heavily shaping teachers’ attitudes and behaviors, thus forming the sociocultural framework for the generation and expression of teachers’ emotions. The sociocultural dimension is a subtle and hidden factor in the formation of teachers’ emotions.

## 6 Conclusion

In summary, the factors influencing teachers’ emotions are both explicit and implicit, internal and external, direct and indirect, micro and macro. The formation of teachers’ emotions is intricately linked not only to teachers’ internal factors but also to the interplay of factors such as teachers’ work context and social culture. These factors are intertwined and interact, producing the internal logic of teachers’ emotions. The mechanism model depicts the three systems of factors influencing the generation of teachers’ emotions and their interconnections. The model can help us to understand the origins and roots of teachers’ emotions and to analyse the interactions between the teachers, their work contexts, and their sociocultural environments. The study will ultimately aid in advancing research on teachers’ emotions.

## 7 Implications

The results of qualitative research are not mechanically created as an integrated whole, but as an intrinsic part of lived experience (Richardson, 2018). Although this study is from China, this model has more general relevance for understanding teachers’ emotions and their pedagogical practices. From a macroscopic perspective, this study helps us to gain a deeper understanding of teachers’ emotions and the visible and invisible mechanisms that generate them. Specifically, administrators can employ this framework to understand the factors influencing and interplaying with teachers’ emotions, prompting reflections on system regulation and school culture construction, with the aim of cultivating a conducive atmosphere for teachers’ professional development. And teachers can use this framework to gain a deeper understanding of their own emotional experience. Moreover, researchers can use this framework to identify the sources of teachers’ emotions, gaining inspiration to conduct in-depth research on these influences and explore the pedagogical implications of teachers’ emotions.

## 8 Limitations and future directions

There are some limitations in our study. First, the sample was exclusively drawn from China, potentially introducing cultural bias and limiting the generalisability of the findings. Moreover, the emotional experiences in this study were mainly confined to six common types—happiness, guilt, worry, fear, complaint, and anger—rather than other complex emotions. The study focused on the emotional experiences of class supervisors and pre-service teachers, neglecting other categories of teachers. Future researchers can expand the study sample to explore the emotional experiences of teachers from different countries, focus on a wider array of emotional experiences, and explore the emotional experiences of teachers with different identities. Such expansions of this research would provide a deeper understanding of the factors and generative mechanisms that influence teachers’ emotions.

## Data availability statement

The original contributions presented in the study are included in the article/supplementary material, further inquiries can be directed to the corresponding authors.

## Ethics statement

The studies involving humans were approved by the Ethics Review Committee of Education School, Guangzhou University. The studies were conducted in accordance with the local legislation and institutional requirements. The participants provided their written informed consent to participate in this study.

## Author contributions

LL: Writing – original draft, Writing – review & editing. WS: Visualization, Writing – review & editing. YX: Supervision, Writing – review & editing. JZ: Resources, Writing – review & editing.

## References

[ref1] Bascia N. Hargreaves A. (Eds.) (2000). The sharp edge of educational change: teaching, leading, and the realities of reform. London, New York: Routledge.

[ref2] Bronfenbrenner U. (1979). The ecology of human development: Experiments by nature and design. Cambridge, Mass: Harvard University Press.

[ref9001] Bingham A. J. (2023). From data management to actionable findings: A five-phase process of qualitative data analysis. Int. J. Qual. Methods. 22, 1–11. doi: 10.1177/16094069231183620

[ref3] Burić I. (2019). The role of emotional labor in explaining teachers’ enthusiasm and students’ outcomes: a multilevel mediational analysis. Learn. Individ. Differ. 70, 12–20. doi: 10.1016/j.lindif.2019.01.002

[ref4] Burić I. Slišković A. Sorić I. (2020). Teachers’ emotions and self-efficacy: a test of reciprocal relations. Front. Psychol. 11:1650. doi: 10.3389/fpsyg.2020.01650, PMID: 32982815 PMC7485560

[ref5] Chen J. (2016). Understanding teacher emotions: the development of a teacher emotion inventory. Teach. Teach. Educ. 55, 68–77. doi: 10.1016/j.tate.2016.01.001

[ref6] Chen J. (2019). Exploring the impact of teacher emotions on their approaches to teaching: a structural equation modelling approach. Br. J. Educ. Psychol. 89, 57–74. doi: 10.1111/bjep.12220, PMID: 29603123

[ref7] Coppola C. Di Martino P. Mollo M. Pacelli T. Sabena C. (2013). Pre-service primary teachers’ emotions: the math-redemption phenomenon., in proceedings of the 37th conference of the International Group for the Psychology of mathematics education, (Germany), 225–232.

[ref8] Coppola C. Di Martino P. Pacelli T. Sabena C. (2012). Primary teachers’ affect: a crucial variable in the teaching of mathematics. Nord. Mat. 17, 101–118.

[ref9] Cross D. I. Hong J. Y. (2012). An ecological examination of teachers’ emotions in the school context. Teach. Teach. Educ. 28, 957–967. doi: 10.1016/j.tate.2012.05.001

[ref10] Cubukcu F. (2013). The significance of teachers’ academic emotions. Procedia-Soc. Behav. Sci. 70, 649–653. doi: 10.1016/j.sbspro.2013.01.105

[ref11] De Ruiter J. A. Poorthuis A. M. G. Koomen H. M. Y. (2021). Teachers’ emotional labor in response to daily events with individual students: the role of teacher–student relationship quality. Teach. Teach. Educ. 107:103467. doi: 10.1016/j.tate.2021.103467

[ref12] Di Martino P. Sabena C. (2011). “Elementary pre-service teachers’ emotions: shadows from the past to the future” in Current state of research on mathematical beliefs XVI. ed. Kislenko K. (Tallinn: Tallinn University), 89–105.

[ref13] Evans D. Butterworth R. Law G. U. (2019). Understanding associations between perceptions of student behaviour, conflict representations in the teacher-student relationship and teachers’ emotional experiences. Teach. Teach. Educ. 82, 55–68. doi: 10.1016/j.tate.2019.03.008

[ref14] Gard M. G. Kring A. M. (2007). Sex differences in the time course of emotion. Emotion 7, 429–437. doi: 10.1037/1528-3542.7.2.429 17516819

[ref15] Givon E. Berkovich R. Oz-Cohen E. Rubinstein K. Singer-Landau E. Udelsman-Danieli G.. (2023). Are women truly “more emotional” than men? Sex differences in an indirect model-based measure of emotional feelings. Curr. Psychol. 42, 32469–32482. doi: 10.1007/s12144-022-04227-z

[ref16] Hammarberg K. Kirkman M. De Lacey S. (2016). Qualitative research methods: when to use them and how to judge them. Hum. Reprod. 31, 498–501. doi: 10.1093/humrep/dev334 26759142

[ref17] Hargreaves A. (2001). The emotional geographies of teachers’ relations with colleagues. Int. J. Educ. Res. 35, 503–527. doi: 10.1016/S0883-0355(02)00006-X

[ref18] Hargreaves A. (2005). Educational change takes ages: life, career and generational factors in teachers’ emotional responses to educational change. Teach. Teach. Educ. 21, 967–983. doi: 10.1016/j.tate.2005.06.007

[ref19] Hepburn S.-J. Carroll A. McCuaig-Holcroft L. (2021). A complementary intervention to promote wellbeing and stress management for early career teachers. Int. J. Environ. Res. Public Health 18:6320. doi: 10.3390/ijerph18126320, PMID: 34207970 PMC8296157

[ref20] Hodgen J. Askew M. (2007). Emotion, identity and teacher learning: becoming a primary mathematics teacher. Oxf. Rev. Educ. 33, 469–487. doi: 10.1080/03054980701451090

[ref21] Isenbarger L. Zembylas M. (2006). The emotional labour of caring in teaching. Teach. Teach. Educ. 22, 120–134. doi: 10.1016/j.tate.2005.07.002

[ref22] Jacobson M. J. Levin J. A. Kapur M. (2019). Education as a complex system: conceptual and methodological implications. Educ. Res. 48, 112–119. doi: 10.3102/0013189X19826958

[ref23] Jiang J. Vauras M. Volet S. Salo A.-E. (2019). Teacher beliefs and emotion expression in light of support for student psychological needs: a qualitative study. Educ. Sci. 9:68. doi: 10.3390/educsci9020068

[ref24] Keller M. M. Chang M.-L. Becker E. S. Goetz T. Frenzel A. C. (2014). Teachers’ emotional experiences and exhaustion as predictors of emotional labor in the classroom: an experience sampling study. Front. Psychol. 5:1442. doi: 10.3389/fpsyg.2014.01442, PMID: 25566124 PMC4263074

[ref25] Lakshman M. Sinha L. Biswas M. Charles M. Arora N. K. (2000). Quantitative vs qualitative research methods. Indian J. Pediatr. 67, 369–377. doi: 10.1007/BF02820690 10885211

[ref26] Luo Z. Yu Q. (2016). A study of teacher identity and teacher emotions in curriculum reforms. Teach. Adm. 6, 8–11.

[ref27] Mevarech Z. R. Maskit D. (2015). The teaching experience and the emotions it evokes. Soc. Psychol. Educ. 18, 241–253. doi: 10.1007/s11218-014-9286-2

[ref28] Nias J. (1996). Thinking about feeling: the emotions in teaching. Camb. J. Educ. 26, 293–306. doi: 10.1080/0305764960260301

[ref29] Reinhard P. Lisa L.-G. (Eds.) (2014). International handbook of emotions in education. New York: Routledge, Taylor & Francis Group.

[ref30] Reio T. G. (2011). “Teacher emotions and socialization-related learning in the context of educational change” in New understandings of teacher’s work: Emotions and educational change. eds. Day C. Lee J. C. (Berlin: Springer), 105–118.

[ref31] Richardson J. (2018). The discovery of cumulative knowledge: strategies for designing and communicating qualitative research. Account. Audit. Account. J. 31, 563–585. doi: 10.1108/AAAJ-08-2014-1808

[ref32] Saunders R. (2013). The role of teacher emotions in change: experiences, patterns and implications for professional development. J. Educ. Change 14, 303–333. doi: 10.1007/s10833-012-9195-0

[ref33] Schutz P. A. Zembylas M. (Eds.) (2009). Advances in teacher emotion research: The impact on teachers’ lives. Boston, MA: Springer US.

[ref34] Smeyers P. (Ed.) (2018). International handbook of philosophy of education. Cham: Springer International Publishing.

[ref35] Strauss A. L. Corbin J. M. (1998). Basics of qualitative research: Techniques and procedures for developing grounded theory. 2nd Edn. Thousand Oaks: Sage Publications.

[ref36] Sun C. Lee J. C. K. (2014). The formation of teachers’ emotions: an ecological perspective. Glob. Educ. 43:82.

[ref37] Sutton R. E. Wheatley K. F. (2003). Teachers’ emotions and teaching: a review of the literature and directions for future research. Educ. Psychol. Rev. 15, 327–385. doi: 10.1023/A:1026131715856

[ref38] Taxer J. L. Becker-Kurz B. Frenzel A. C. (2019). Do quality teacher–student relationships protect teachers from emotional exhaustion? The mediating role of enjoyment and anger. Soc. Psychol. Educ. 22, 209–226. doi: 10.1007/s11218-018-9468-4

[ref39] Tian B. Li L. (2006). The influence of school organizational climate on job burnout. J. Psychol. Sci. 29, 189–193. doi: 10.16719/j.cnki.1671-6981.2006.01.052

[ref40] Van Manen M. (1982). Phenomenological pedagogy. Curric. Inq. 12, 283–299. doi: 10.1080/03626784.1982.11075844

[ref41] Van Veen K. Lasky S. (2005). Emotions as a lens to explore teacher identity and change: different theoretical approaches. Teach. Teach. Educ. 21, 895–898. doi: 10.1016/j.tate.2005.06.002

[ref42] Van Veen K. Sleegers P. (2006). How does it feel? Teachers’ emotions in a context of change. J. Curric. Stud. 38, 85–111. doi: 10.1080/00220270500109304

[ref43] Van Veen K. Sleegers P. (2009). “Teachers’ emotions in a context of reforms: to a deeper understanding of teachers and reforms” in Advances in teacher emotion research. eds. Schutz P. A. Zembylas M. (Boston, MA: Springer US), 233–251.

[ref44] Van Veen K. Sleegers P. Van De Ven P.-H. (2005). One teacher’s identity, emotions, and commitment to change: a case study into the cognitive–affective processes of a secondary school teacher in the context of reforms. Teach. Teach. Educ. 21, 917–934. doi: 10.1016/j.tate.2005.06.004

[ref45] Wang J. Cheng L. (2011). Hargreaves’ views of teachers and teaching. Glob. Educ. 40, 15–21.

[ref46] Wu Y. Wang S. Nie Y. Zhao L. (2010). Modern university management: from institutional norms to cultural immersion. J. Natl. Acad. Educ. Adm. 28, 29–32.

[ref47] Yin H. (2007). Teacher emotion: An issue urgently to be taken into consideration. Res. Educ. Dev. 6, 44–48.

[ref48] Yuan X. (2019). A survey of emotional intelligence of primary school teachers under demographic variables. J. Jilin Prov. Inst. Educ. 35, 110–114. doi: 10.16083/j.cnki.1671-1580.2019.11.021

[ref49] Zembylas M. (2007). Emotional ecology: the intersection of emotional knowledge and pedagogical content knowledge in teaching. Teach. Teach. Educ. 23, 355–367. doi: 10.1016/j.tate.2006.12.002

[ref50] Zhang J. (2007). On the professional identity of teachers. Res. Educ. Dev. 46, 39–41.

[ref51] Zhou J. (2020). An empirical study of strategies and factors affecting emotional labour of high school teachers. Eachers J. 7, 66–70.

